# Predicting Sit-to-Stand Adaptations due to Muscle Strength Deficits and Assistance Trajectories to Complement Them

**DOI:** 10.3389/fbioe.2022.799836

**Published:** 2022-03-18

**Authors:** Vinay Kumar, Takahide Yoshiike, Tomohiro Shibata

**Affiliations:** ^1^ Department of Human Intelligence Systems, Graduate School of Life Science and Systems Engineering, Kyushu Institute of Technology, Kitakyushu, Japan; ^2^ Honda R&D Co., Ltd., Saitama, Japan

**Keywords:** sit-to-stand, musculoskeletal model, strength deficit, single shooting optimization, open loop controller, assist-as-needed

## Abstract

Sit-to-stand (STS) transition is one of the most bio-mechanically challenging task necessary for performing activities of daily life. With muscle strength being the most dominant, many co-occurring factors influence how individuals perform STS. This study investigates the STS changes and STS failure caused by strength deficits using the trajectories generated employing an open-loop single shooting optimization framework and musculoskeletal models. The strength deficits were introduced by simultaneously scaling the maximum isometric strength of muscles in steps of 20%. The optimization framework could generate successful STS transitions for models with up to 60% strength deficits. The joint angle kinematics, muscle activation patterns, and the ground reaction forces from the 0% strength deficit model’s STS transition match those observed experimentally for a healthy adult in literature. Comparison of different strength deficit STS trajectories shows that the vasti muscle saturation leads to reduced activation of the antagonistic hamstring muscle, and consequently, the gluteus maximus muscle saturation. Subsequently, the observation of reduced hamstring activation and the motion tracking results are used to suggest the vasti muscle weakness to be responsible for STS failure. Finally, the successful STS trajectory of the externally assisted 80% strength deficit model is presented to demonstrate the optimization framework’s capability to synthesize assisted STS transition. The trajectory features utilization of external assistance as and when needed to complement strength deficits for successful STS transition. Our results will help plan intervention and design novel STS assistance devices.

## 1 Introduction

Sit-to-stand (STS) transition is a precursor to walking, hence critical for performing daily life activities and an independent lifestyle. Lower extremity strength plays an important role in human STS, and its deficits are thought to limit the STS functionality. Studies have shown that the lower extremity strength is a strong predictor of the ability of older adults to perform STS from the lowest possible chair height (Hughes et al., 1996; Schenkman et al., 1996). This study aims to identify the STS changes and the STS failure caused by lower extremity strength deficits and the external assistance trajectories that can complement them for successful STS transition.

The decline in muscle strength often co-occurs with other physiological and psychological impediments such as reduced balance, joint pain, and depression, making it difficult to access its independent effect on STS using experiments (Lord et al., 2002). Also, besides subject-specific factors, STS is influenced by many extrinsic factors like foot placement, knee position, and chair height, making designing and conducting experiments complex. Some past studies have used STS trajectories generated using optimization and human models to avoid the complications of experiments. Pandy et al. (1995) presented a cost function that generates STS trajectories with similar muscle activations to those of experiments. Bobbert et al. (2016) and Yokota et al. (2016) searched for trajectories that reduced loads on the muscles and the knee joint. However, the studies mentioned above have made either minimal or no observations about STS changes caused by strength deficits. Further, these studies have also not investigated how strength deficits might lead to unsuccessful STS.

Many older individuals incapable of independent STS transition can perform the same when assisted externally. This external assistance can help maintain or recover lower extremity strength when provided in an assist-as-needed manner. Thus it is desirable to generate reference assistance trajectories that assist as and when needed and by the amount that is needed. Mombaur and Hoang (2017) and Geravand et al. (2017) have used optimization to discover assistance trajectories that support part of the user’s weight during STS and squat-to-stand motions, respectively. However, both the studies use human models with independently torque actuated joints. The hamstrings and the rectus femoris are two biarticular muscles that play an essential role in the STS transition. Their biarticularity couples the torques produced at the hip and knee joints. This coupling should not be ignored, especially when generating reference STS assistance trajectories, as it may lead to assistance profiles that over actuates one of these muscles, leading to muscle contracture and eventually lower back issues. The coupling is also crucial for accurately investigating the STS changes and the STS failure caused by the strength deficits. Thus, musculoskeletal models with varying degrees of strength deficits are used in this study to generate assisted and unassisted STS trajectories.

In this study, the single shooting optimization framework used to generate STS trajectories is detailed first, followed by the tools used to analyze them. Subsequently, the joint angle, the muscle activation, and the ground and seat reaction force patterns from the 0% strength deficit model’s STS trajectory are contrasted against those observed experimentally for a healthy adult in literature for validation. Then, the different strength deficit trajectories are compared to observe the STS changes caused by strength deficits, followed by an investigation of the STS failure using motion-tracking results. Finally, the successful STS trajectory of an externally assisted musculoskeletal model, incapable of performing unaided STS transition, is presented to demonstrate the optimization framework’s ability to generate externally assisted STS trajectory. The findings of this study will help plan intervention and design novel STS assistance devices that operate in an assist-as-needed manner.

Within the single shooting optimization framework, we have parameterized the open-loop excitation trajectories of the actuators similarly to Pandy et al. (1995), and Yokota et al. (2016). The excitation trajectories are used to integrate the system’s equation of motion of the equation forward in time to generate the resultant motion. The cost function evaluated on the resultant motion is then used to tune the actuator’s excitation trajectories. Another possible optimization framework’s structure is in whom the optimization is performed over the joint angle space. The tuning of joint angle trajectories is based on the solutions of inverse dynamics for skeletal models and the solutions of inverse dynamics and static optimization for musculoskeletal models. Such frameworks are used for STS synthesis in Sadeghi et al. (2013); Norman-Gerum and McPhee (2018); Yang and Ozsoy (2020), to discover STS trajectories with minimum actuator efforts in Yoshioka et al. (2007, 2012), and to predict the unilateral grab-rail assisted STS trajectories of a virtually unhealthy adult in Yang and Ozsoy (2021); Ozsoy and Yang (2021). Direct collocation is another potential optimization framework. This framework performs optimization over both the joint angle and the actuator excitation space (Bobbert et al., 2016). We selected open-loop single shooting trajectory optimization for its straightforward implementation and effortless extension to incorporate closed-loop controllers in future works.

It is difficult to identify and detail all of the parameters that shape the STS trajectories generated using optimization. For example, Bobbert et al. (2016), and Yokota et al. (2016) does not contain information about the initial guesses to the optimization algorithm, while Pandy et al. (1995) does not include information about the mechanical limits used to restrict the motion to the physiologically plausible range. Therefore we have made all the source code and results from this study public at https://github.com/ShibataLab/PredictiveSTS.

## 2 Methods

**FIGURE 1:**
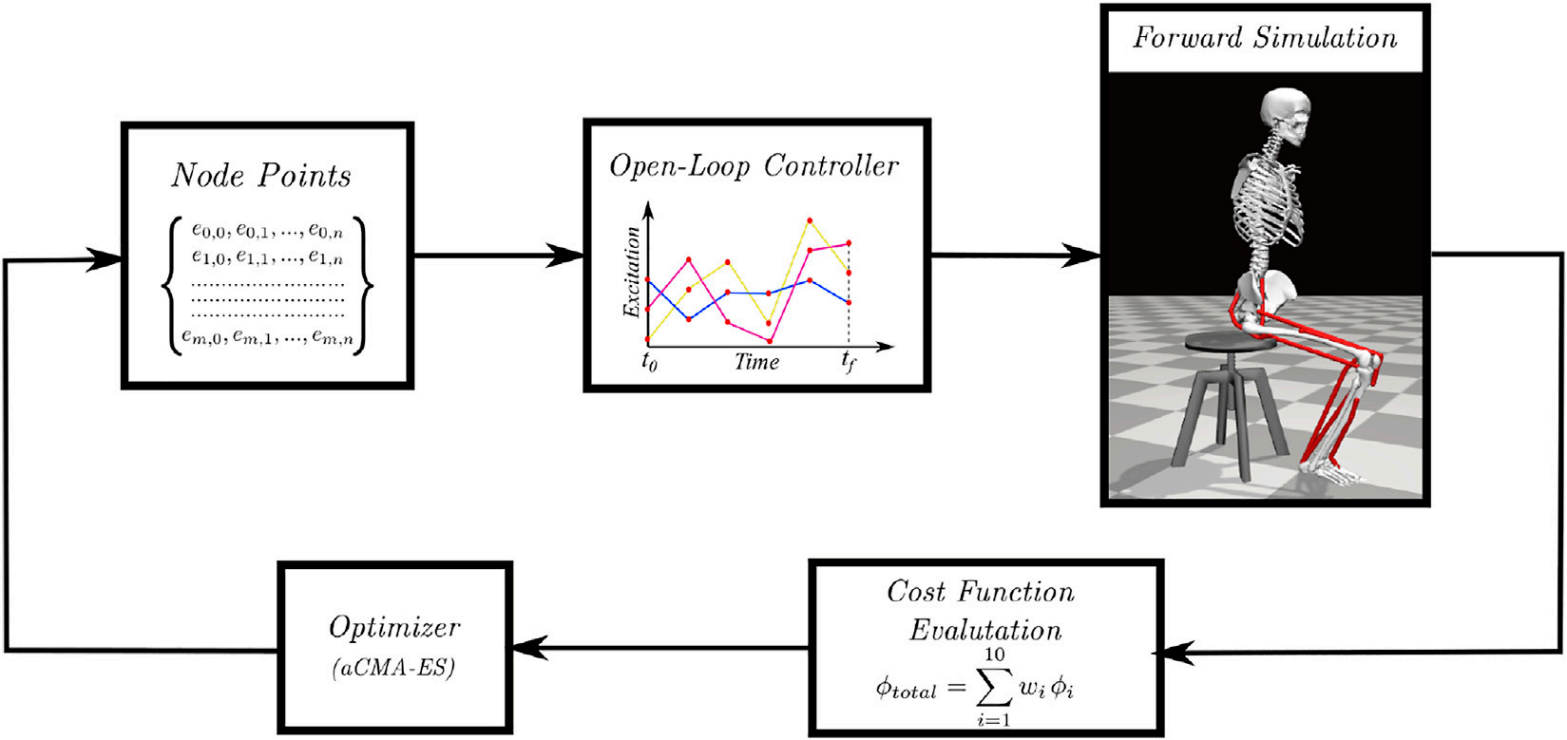
Overview of single shooting optimization framework. The red dots in the open-loop controller represents the node points obtained from the discretization of excitation trajectories.

An overview of the single shooting optimization framework used to generate STS trajectories in this study is shown in Figure 1. The framework tunes the values of decision variables using the aCMA-ES algorithm (Arnold and Hansen, 2010). aCMA-ES is a stochastic gradient-free optimization algorithm that adapts a Gaussian distribution towards low energy regions. It was selected for its enhanced robustness to locally optimal solutions compared to the gradient-based algorithms. At each generation, aCMA-ES samples a batch of candidate solutions from the Gaussian being adapted. Subsequently, the cost function values are evaluated for all the candidates on the respective forward simulations. aCMA-ES then adapts the Gaussian based on the cost function values and samples the next batch of candidate solutions and so on until one of the stopping criteria is met.

In subsection 2.1, the musculoskeletal models used with the optimization framework to generate STS trajectories are detailed. Subsection 2.2 includes the details of the decision variables and the termination criteria used with the optimization framework, while subsection 2.3 describes the cost function. Subsection 2.4 includes a summary of the motion-tracking setup used to investigate the STS failure. The final subsection 2.5, details the steps used to process the experimental data against whom the 0% strength deficit model’s STS trajectory is validated. We refer to the STS trajectories as the model’s trajectories for conciseness, even though the models were only a single component of the optimization framework.

### 2.1 Musculoskeletal Model

**FIGURE 2:**
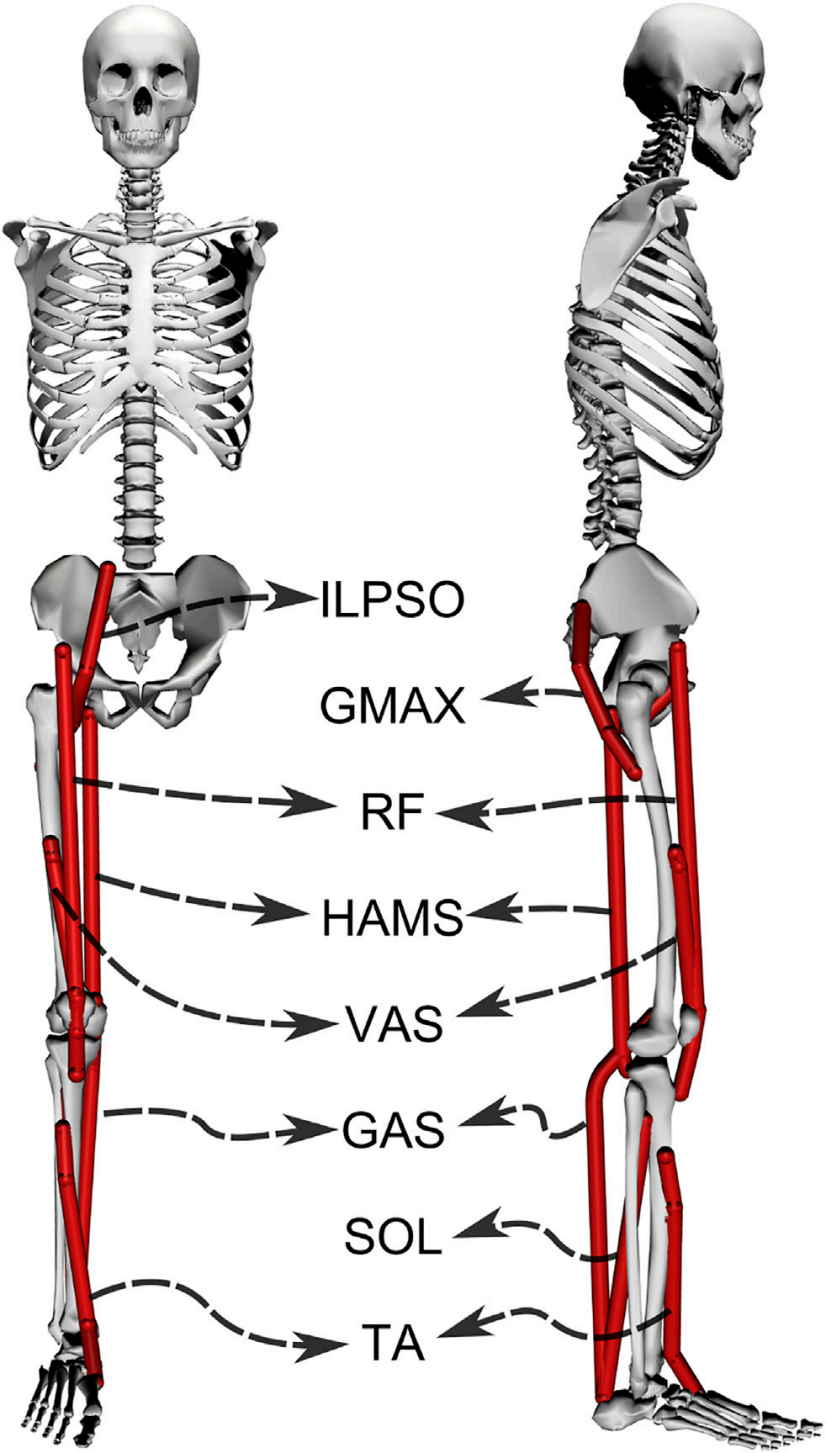
A planar musculoskeletal model for sit-to-stand. The model’s musculotendon actuators (red lines) represents the major uniarticular and biarticular muscle groups that drive the sit-to-stand motion in the sagittal plane, i.e., iliopsoas (ILPSO), gluteus maximus (GMAX), biarticular rectus femoris (RF), biarticular hamstrings (HAMS), vasti (VAS), gastrocnemius (GAS), soleus (SOL), and tibialis anterior (TA). The model has three degrees of freedom distributed at the hip, knee and ankle joints.

Musculoskeletal models with different strength deficits for this study were obtained by simultaneously scaling the maximum isometric strengths of the muscles present within the base model. The base model, also shown in Figure 2, is a simplified version of the LaiArnold2017 model (Lai et al., 2017). The LaiArnold2017 model represents an average-sized adult male of mass 75 *Kg* and height 170 *cm*. The base model is 2D with eight hill-type muscles and three degrees of freedom, while the source model is 3D with 80 hill type muscles and 37 degrees of freedom. The simplifications were needed to make the optimization problem computationally tractable. The following paragraphs detail some of these simplifications along with other modelling details.

From the LaiArnold2017 model, the left leg and the associated muscles were removed. The masses of arms, forearms, hands and the head were lumped to the torso’s center of mass (COM). The mass and inertia of the torso after lumping were halved to account for the missing left leg and the associated muscles. The right foot was fixed to the ground using a weld joint. Then the degrees of freedom corresponding to the sagittal plane motion of the ankle, knee, hip and lumbar joints were added. The 0° angle of the hip, knee, ankle and lumbar joints corresponds model standing upright. From 0°, the positive joint angles correspond to ankle dorsiflexion, knee flexion, hip flexion, and lumbar extension; and the negative joint angles correspond to the opposite. The lumbar joint was locked to −10°, for reasons explained in subsection 2.5 and thus, the model effectively has three degrees of freedom. The lower extremity muscles with similar functions were combined to single muscle-tendon units as realized in Ong et al. (2019). Figure 2 shows insertion points and the paths of the resultant muscles included in the model, i.e., gluteus maximus (GMAX), biarticular hamstrings (HAMS), iliopsoas (ILPSO), biarticular rectus femoris (RF), vasti (VAS), biarticular gastrocnemius (GAS), soleus (SOL), and tibialis anterior (TA). Table 1 lists the maximum isometric strengths for the muscles included in the base model along with the acronyms. At the beginning of simulations, the muscle states were set by equilibrating the muscle-tendon units with the default activation of 0.05.

**TABLE 1:** Muscles included in the model, their acronyms and their respective maximum isometric strengths for the 0% strength deficit model.

Muscle	Acronym	Maximum isometric strength (N)
Iliopsoas	ILPSO	2697.3
Gluteus maximus	GMAX	3337.6
Biarticular rectus femoris	RF	2191.7
Biarticular hamstrings	HAMS	4105.5
Vasti	VAS	9594.0
Biarticular gastrocnemius	GAS	4690.6
Soleus	SOL	7925.0
Tibialis anterior	TA	2116.8

The chair-body contact interactions were modelled using a point on point kinematic constraint between the femur head and the chair. During simulation, the kinematic constraint was disabled if the vertical reaction forces required to maintain it turned non-compressive or satisfied the slipping condition. The seat kinematic constraint, once disabled, could not be re-engaged and thus prevented the optimization from getting stuck into local optima with multiple chair rises. The model had nonlinear torsional springs representing ligaments at the hip, knee, and ankle joints, limiting the motions to physiologically plausible ranges. They generated torques when the hip joint flex beyond 120°or extends below 30°, or the knee joint flex beyond 140° or extend beyond 0°, or the ankle dorsiflex beyond 30° or plantarflex beyond 40°. These ranges are from the LaiArnold2017 model. The remaining torsional spring parameters are from Ong et al. (2019).

External assistance was introduced at the torso’s COM in the musculoskeletal model that failed to perform unassisted STS transition. The rationale behind introducing it at the torso is explained in subsection 3.3. For implementation simplicity, the external assistance was modelled using two independent point forces acting in the vertical and horizontal directions. Their respective magnitudes were limited to the 0–200 *N* range. Before computing actuation, the excitation signals to point forces were passed through first-order activation dynamics. It made the external assistance trajectories smooth and thus reduced the optimization framework’s sensitivity to the values of individual assistance force decision variables. The first-order activation dynamics had a time constant of 0.1 s. The OpenSim API (Delp et al., 2007) was used to formulate the musculoskeletal model’s equation of motion and their forward integration.

### 2.2 Optimization Setup

The optimization framework tuned the STS duration (*t*_*f*_) and the node point values obtained by discretizing the excitation trajectories of the actuators present within the musculoskeletal model. The discretization was performed using piecewise linear functions with a fixed time step of 0.1 s between consecutive nodes. The upper limit for simulation duration (*t*_max_) was selected to be 1.6 s, similar to Yokota et al. (2016). All the musculoskeletal models had eight hill-type muscles, and the externally assisted musculoskeletal model had two additional point actuators. At *t*_0_, the actuators had their default activation. Thus, the optimization problem had 129 decision variables when generating unassisted STS trajectories and 161 decision variables when generating assisted STS trajectories.

As mentioned before, aCMA-ES is a stochastic gradient-free optimization algorithm that adapts a Gaussian distribution towards low energy regions. The node point values corresponding to the model sitting in a chair were used as the initial guess for the mean of the Gaussian. The algorithm was restarted if the number of generations exceeded 4,000 or if the improvement in the cost values was lower than 1.0 for the best candidate solutions over the immediate 250 generations. At each restart, the generation counter and the covariance matrix were reset to default, and the mean was set to the been-seen candidate solution till then. Four restarts were performed to account for the stochasticity of the optimization algorithm and the non-linearity optimization space before selecting the optimal candidate solution. We used the *libcmaes* library (CMA-ES, 2013) for the aCMA-ES algorithm.

### 2.3 Cost Function

The cost function we selected to engender STS transition is a linear combination of ten different terms and can be expressed as follows:

$$\phi_{total}=\overset{10}{\underset{i=1}{\sum }}w_{i}\phi_{i}$$ (1)

where *w*_*i*_ is the relative weight of *i*th cost term, i.e., *ϕ*_*i*_. The mathematical expressions for the ten cost terms are given in Eqs 2–12. Please refer to Table 2 for the list of symbols used in these equations. All the elements associated with different costs were computed in SI units.

$$\phi_{1}=\frac{d\left(C_{f},C_{goal}\right)}{d\left(C_{0},C_{goal}\right)}$$ (2)

$$\phi_{2}=\left[1-\alpha \right]\int_{t_{0}}^{t_{f}}\frac{e^{t/\tau }}{\tau \left[e^{t_{f}/\tau }-1\right]}F_{chair,y}\left(t\right)dt$$ (3)

$$\phi_{3}=\sqrt{\frac{\underset{i}{\sum }\int_{t_{0}}^{t_{f}}a_{i}{\left(t\right)}^{2}dt}{\sum i}}$$ (4)

$$\phi_{4}=\sqrt{\frac{\underset{i}{\sum }\int_{t_{0}}^{t_{f}}{{\dot{a}}_{i}\left(t\right)}^{2}dt}{\sum i}}$$ (5)

$$\phi_{5}=\int_{t_{0}}^{t_{f}}\|F_{Assist}\left(t\right)\|dt$$ (6)

$$\phi_{6}=\underset{n}{\sum }\int_{t_{0}}^{t_{f}}|T_{n,limit}\left(t\right)|dt$$ (7)

$$\phi_{7}=\alpha \;\underset{\left\{t_{0},t_{f}\right\}}{\max}\left(0,|F_{feet,x}\left(t\right)|-\mu F_{feet,y}\left(t\right)\right)$$ (8)

$$\phi_{8}=\alpha \;\underset{\left\{t_{0},t_{f}\right\}}{\max}|ZMP_{x}\left(t\right)-Feet_{x}\left(t\right)|dt$$ (9)

$$\phi_{9}=\alpha \;\left[\left|{\dot{\theta }}_{hip}\left(t_{f}\right)|+|{\dot{\theta }}_{knee}\left(t_{f}\right)|+|{\dot{\theta }}_{ankle}\left(t_{f}\right)\right|\right]$$ (10)

$$\phi_{10}=\alpha \left[\begin{array}{cc} |\underset{\left\{t_{SR},t_{f}\right\}}{\max}\left(F_{feet,y}\left(t\right)\right)-mg|+ & \\ |\underset{\left\{t_{SR},t_{f}\right\}}{\min}\left(F_{feet,y}\left(t\right)\right)-mg|+ & \\ \left|F_{feet,y}\left(t_{f}\right)-mg\right| \end{array}\right]$$ (11)

$$\alpha =1-\frac{\min\left(d\left(C_{f},C_{goal}\right),d\left(C_{0},C_{goal}\right)\right)}{d\left(C_{0},C_{goal}\right)}$$ (12)

Cost *ϕ*_1_ is the ratio of euclidean distances between the goal and *t*_*f*_ COM positions, and the goal and *t*_0_ COM positions. The goal COM position corresponds to the model standing upright. Cost *ϕ*_2_ penalizes the model staying in contact with the chair. Cost *ϕ*_2_ features an increasing exponential and thus penalizes the chair contact interactions more during the later part of simulation than prior. Costs *ϕ*_3_ and *ϕ*_4_ penalize the control effort and its rate of change, respectively. Cost *ϕ*_5_ demotivates excessive use of external assistance. It was set to zero for the unassisted STS trajectories. Cost *ϕ*_6_ discourages hyper-flexion and hyper-extension of joints. Costs *ϕ*_7_ and *ϕ*_8_ respectively penalize the feet contact forces that would lead to slip or tipping over the heel or toes. Cost *ϕ*_9_ penalizes the body motion at *t*_*f*_ while cost *ϕ*_10_ penalizes the excessive body accelerations.

**TABLE 2:** List of symbols.

Variable	Description
*t*	Time
‥(*t*)	Value of a expression ‥ at time *t*
|‥|	The absolute value expression ‥
*t*_0_	Simulation start time
*t*_*f*_	Simulation final time
*t*_max_	Upper limit of *t*_*f*_
*t*_*SR*_	Time of seat release
*C*_0_	Center of mass position at *t*_0_
*C*_*f*_	Center of mass position at *t*_*f*_
*C*_*goal*_	Center of mass position for standing posture
*d* (*C*_1_, *C*_2_)	Euclidean distance between center of mass positions at *t*_1_ and *t*_2_
*α*	% Sit to stand completion
*F*_*chair*,*y*_	*y* component of constraint force applied by the chair on the femur head
*τ*	Time constant
*a*_*i*_	Activation of actuator *i*
‖*F*_*Assist*_‖	Magnitude of external assistance
*T*_*n*,*limit*_	Torque generated by the torsional limit spring at the *n*th joint
*F*_*feet*,*n*_	Component of force applied along *n* direction by the ground on the feet
*ZMP*_*x*_	*x* coordinate of feet force zero moment point
$\dot{\theta_{j}}$	Velocity of joint *j*
*Feet*_*x*_	*x* coordinate of the mid point between heel and toes
*mg*	Weight of musculoskeletal model

**FIGURE 3:**
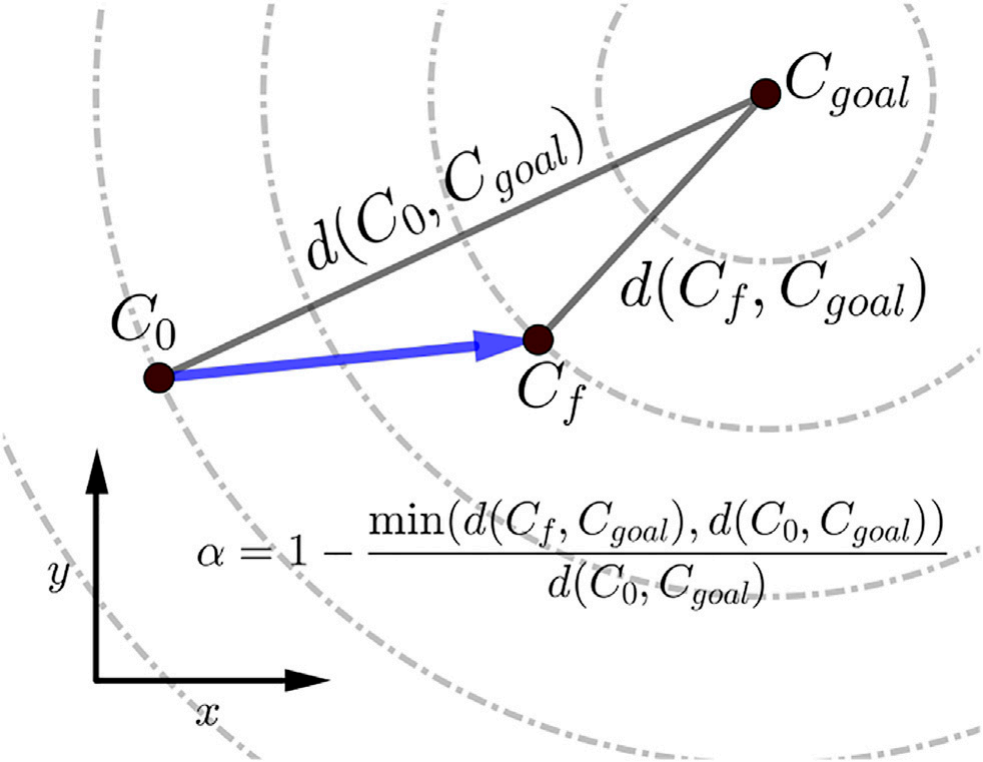
Scalar *α*, used within the cost expressions, represents the percentage of STS completion and ranges between 0 and 1. The dashed circles show the states that are equidistant from the *C*_*goal*_.

**FIGURE 4:**
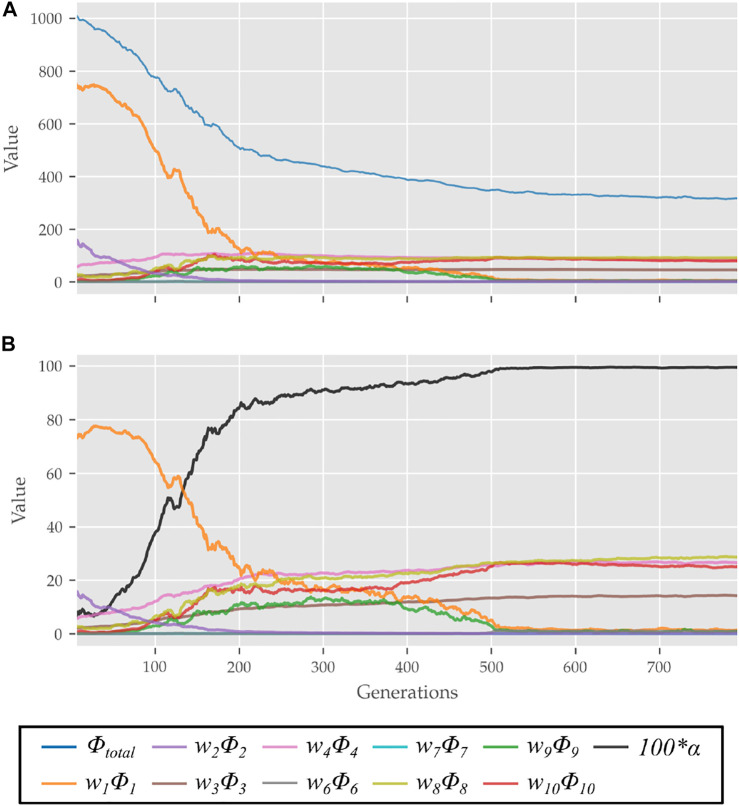
**(A)** Evolution of different costs and **(B)** their relative contributions to the total cost for the best candidates observed during optimization using the 0% strength deficit model. The costs were smoothed using a rolling average of 10 generations for this plot.

The scalar *α* represents STS progress and is illustrated in Figure 3. While learning to perform STS, the optimization first comes across unstable trajectories. Costs *ϕ*_7_ to *ϕ*_10_ are scaled by *α* to prevent them from hindering the exploration of unstable STS trajectories for stable ones. It can be seen in Figure 4 that during the initial generations, the value of *α* is closer to zero as *C*_*f*_ is far away from *C*_*goal*_. Then as the optimization progresses, cost *ϕ*_2_ moves the model out chair and cost *ϕ*_1_ moves it towards standing posture. This moves *C*_*f*_ towards *C*_*goal*_, and the value of *α* and so the contribution costs *ϕ*_7_ to *ϕ*_10_ increases. As the model learns to stand up, an increasing amount of control effort is required and thus, the relative contributions of costs *ϕ*_3_ and *ϕ*_4_ increase with optimization progress. The values of relative weights associated with different costs, i.e., *w*_*i*_, were determined by trial and error and listed in Table 3 along with other cost function related hyperparameters. Supplementary Figure S1 of the supplementary material shows the generated STS trajectories are reasonably robust to the *w*_*i*_ values.

**TABLE 3:** Cost function hyperparameters.

Variable	Value
*τ*	*t*_max_/8
*w*_1_	800
*w*_2_	1.2
*w*_3_	175
*w*_4_	70
*w*_5_	5
*w*_6_	10
*w*_7_	0.1
*w*_8_	1,000
*w*_9_	6
*w*_10_	0.3

### 2.4 Motion Tracking Setup

The OpenSim CMC tool-based motion tracking was used to investigate the STS failure in this study. The CMC tool computes the actuator excitation levels at user-specified time intervals that will drive the generalized coordinates $\left(\vec{q}\right)$ of the musculoskeletal model towards a desired kinematic trajectory $\left({\vec{q}}_{\exp}\right)$ in the presence of external forces. At any given time *t*, the CMC tool first computes the desired acceleration ${\ddot{\vec{q}}}^{\;*}$ using the following proportional derivative control law:

$${\ddot{\vec{q}}}^{\;*}\left(t+T\right)={\ddot{\vec{q}}}_{\exp}\left(t+T\right)+{\vec{k}}_{v}\left[{\dot{\vec{q}}}_{\exp}\left(t\right)-\dot{\vec{q}}\left(t\right)\right]+{\vec{k}}_{p}\left[{\vec{q}}_{\exp}\left(t\right)-\vec{q}\left(t\right)\right]$$ (13)

where, ${\vec{k}}_{v}$ and ${\vec{k}}_{p}$ are the feedback gains on the velocity and position errors, respectively. Since the forces that muscles apply cannot change instantaneously, the desired accelerations are computed some small-time *T* in the future. Then, CMC tool uses static optimization to distribute the load across synergistic actuators using static optimization. CMC tool offers two formulations for static optimization referred to as slow target and fast target. We used the fast target formulation. It minimizes the sum of squared controls augmented by a set of equality constraints which can be mathematically represented as follows:

$$J=\underset{i=1}{\sum }e_{i}^{2}$$ (14)

$$C_{j}={\ddot{q}}_{j}^{\;*}-{\ddot{q}}_{j\;}\forall j$$ (15)

where *e*_*i*_ is the control input/excitation of *i*th actuator at time *t* and *q*_*j*_ is the *j*th generalized coordinate. Since for many ${\ddot{q}}_{j}^{\;*}$ the muscles might not be able to produce sufficient forces, ideal torque actuators are added to the musculoskeletal model to prevent the fast target formulation from failing. Usually, the forces/torques produced per unit control effort for the ideal actuators is much lower than muscles. In such setups, following Eq. 14, ideal torque actuators produce significant force/torque only when the muscles are saturated, and hence they are also referred to as reserve actuators. Since the CMC tool does not support event-based disabling of kinematic constraints, the seat forces were computed during the forward simulation and then supplied as external forces.

### 2.5 Experimental Data Processing

We have used the experimental data recordings of Lao et al. (2019) and Lao et al. (2020) to validate the 0% strength deficit model’s STS trajectory generated. The experimental data contains optical marker trajectories, surface EMG signals and the ground and seat-pan reaction forces for 12 healthy adult subjects performing assisted and unassisted STS. Since the experimental data does not contain functional trials needed to scale musculoskeletal models, we have used the recordings of the subject with height and weight closest to our model. The selected subject weighs 71 *Kg* and is 169 *cm* tall. The source musculoskeletal model represents an adult male of mass 75 *Kg* and height 170 *cm*.

**FIGURE 5:**
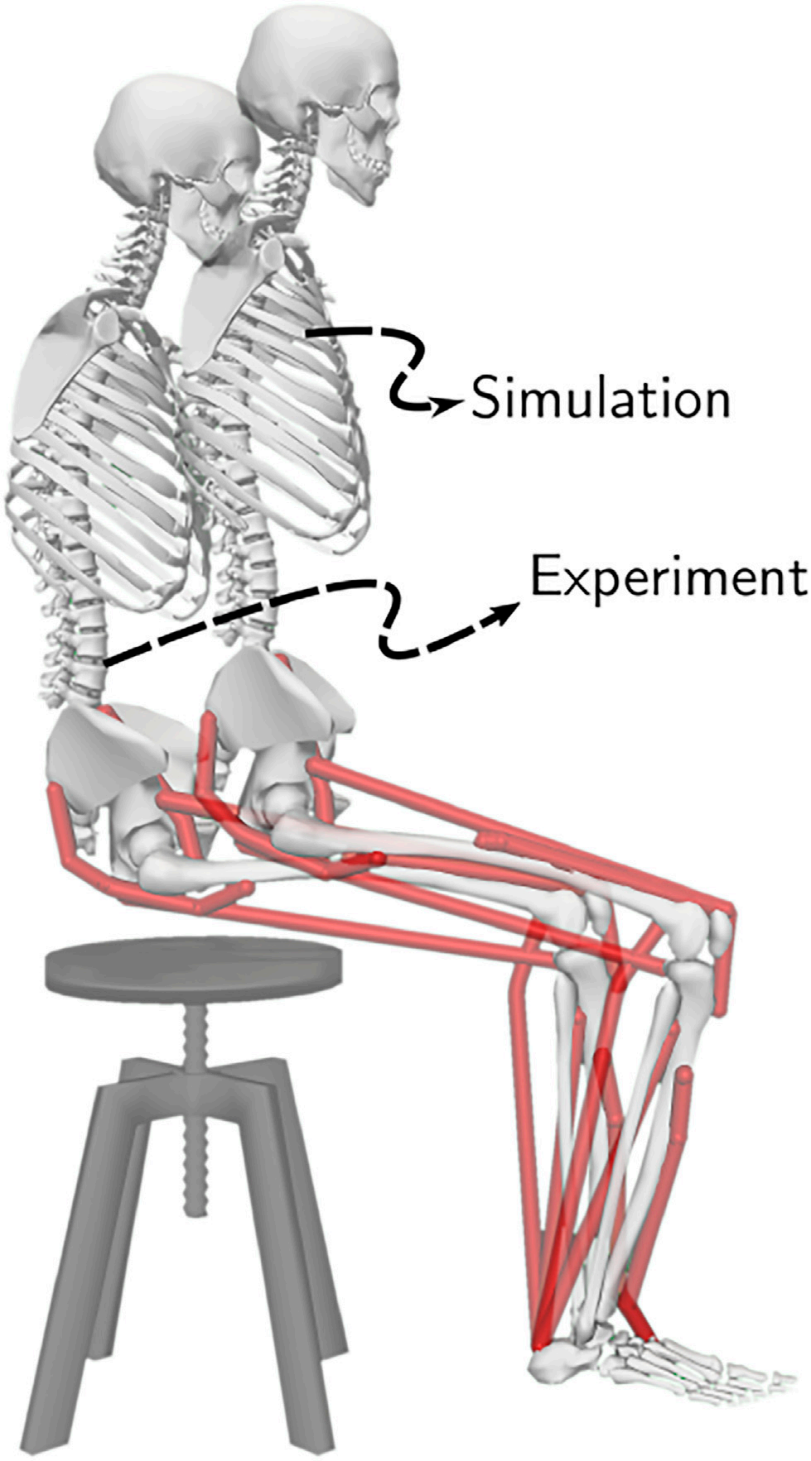
The initial posture used to generate STS trajectories and the mean initial posture observed during experiments. The model was moved slightly forward for simulation to compensate for its non-actuated lumbar joint.

The unassisted STS recordings have six trials under each of four conditions, i.e., arms folded across chest, arms hanging freely next to the body, natural STS, and slow pace imitating assisted STS. We used the 18 trials belonging to the first three categories. The optical markers were fixed to the musculoskeletal model on the average marker positions of the T-pose trial. This musculoskeletal model with registered optical markers was used for inverse kinematics. We defined the beginning and the end of STS as the times when hip flexion and hip extensions velocities smoothed with a rolling window of 0.1 s were respectively higher or lower than 20°/*s*. The resulting joint trajectories from the 18 trials are shown in Supplementary Figure S2. The mean initial posture observed in experiments is compared to the initial posture used to generate STS trajectories in Figure 5. As can be observed, the simulation model was moved slightly forward towards the feet, and the lumbar joint was locked to −10°. The adjustments were made to compensate for the non-actuated lumbar joint. Also, the simulation’s initial posture is easier to stand up from due to the torso lying closer to the feet.

The sEMG signals were processed by first passing through a fourth-order Butterworth bandpass filter with 10 and 350 Hz cutoff frequencies. Then they were rectified and subsequently passed through a fourth-order Butterworth lowpass filter of 3 Hz cutoff frequency. Finally, the signals were normalized using the peak values from the maximum voluntary control trials. The ground and seat reaction force trajectories were not processed. Supplementary Figures S3, S4 respectively illustrate the sEMG and ground and seat reaction force trajectories from the 18 trails used in this study.

## 3 Results

**FIGURE 6:**
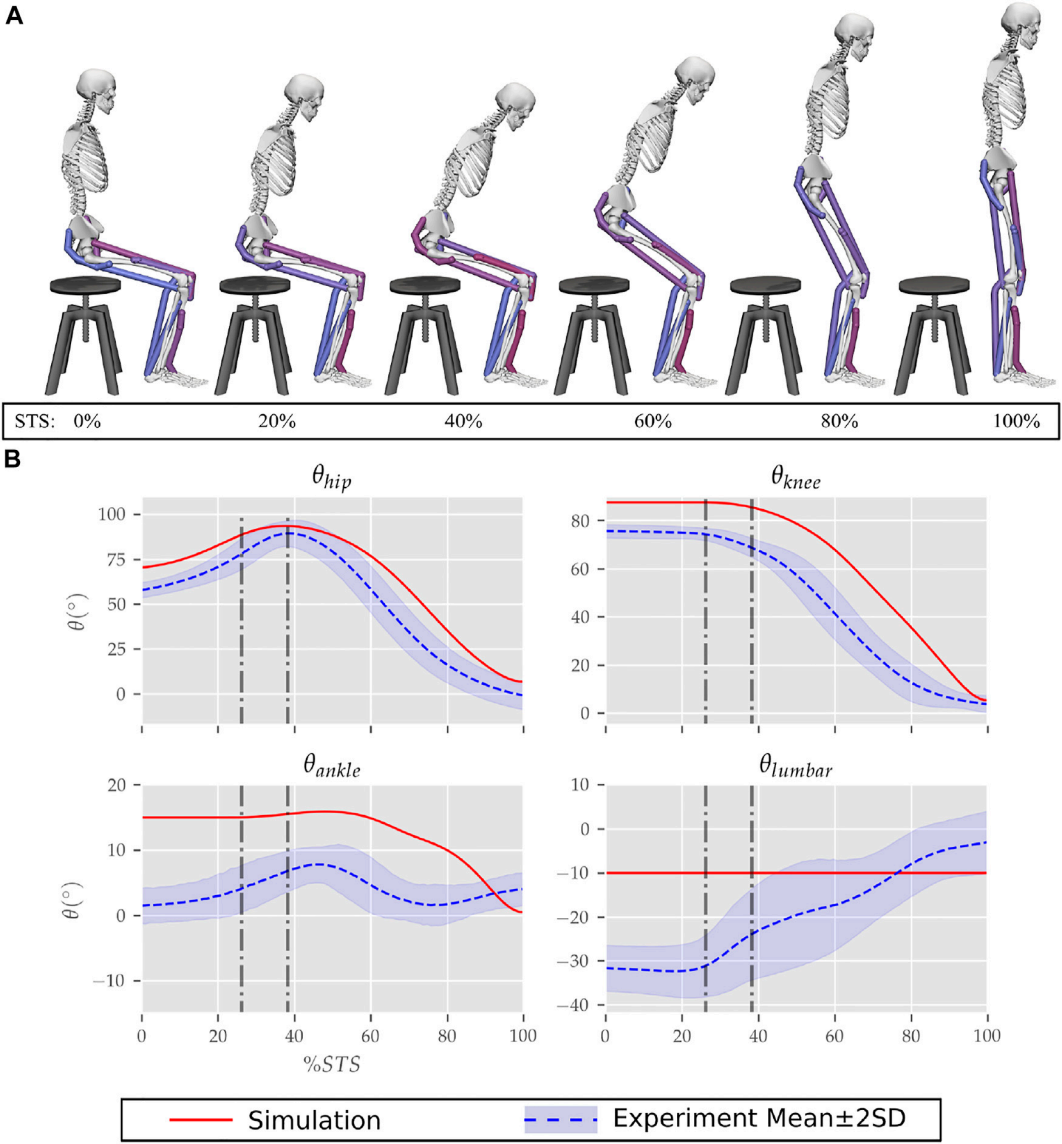
**(A)** Different postures observed during the 0% strength deficit model’s STS transition and **(B)** the comparison of associated joint angle trajectories against experimental observations. The first vertical dotted line marks the point when the model lost contact with the chair, and the second vertical dotted line marks the posture with maximum hip flexion.

**FIGURE 7:**
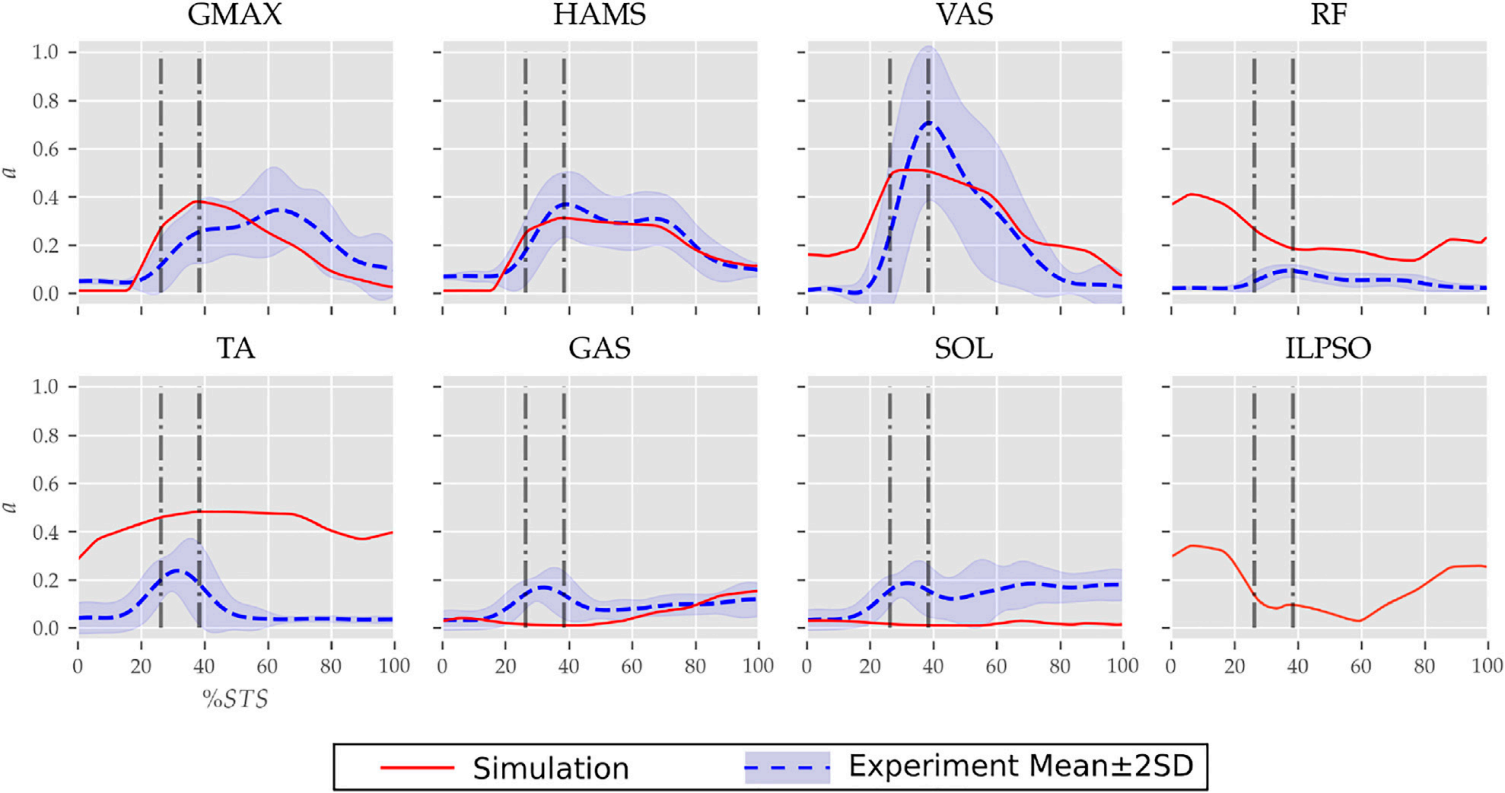
Muscle activation trajectories associated with the 0% strength deficit model’s STS transition and those recorded experimentally.

**FIGURE 8:**
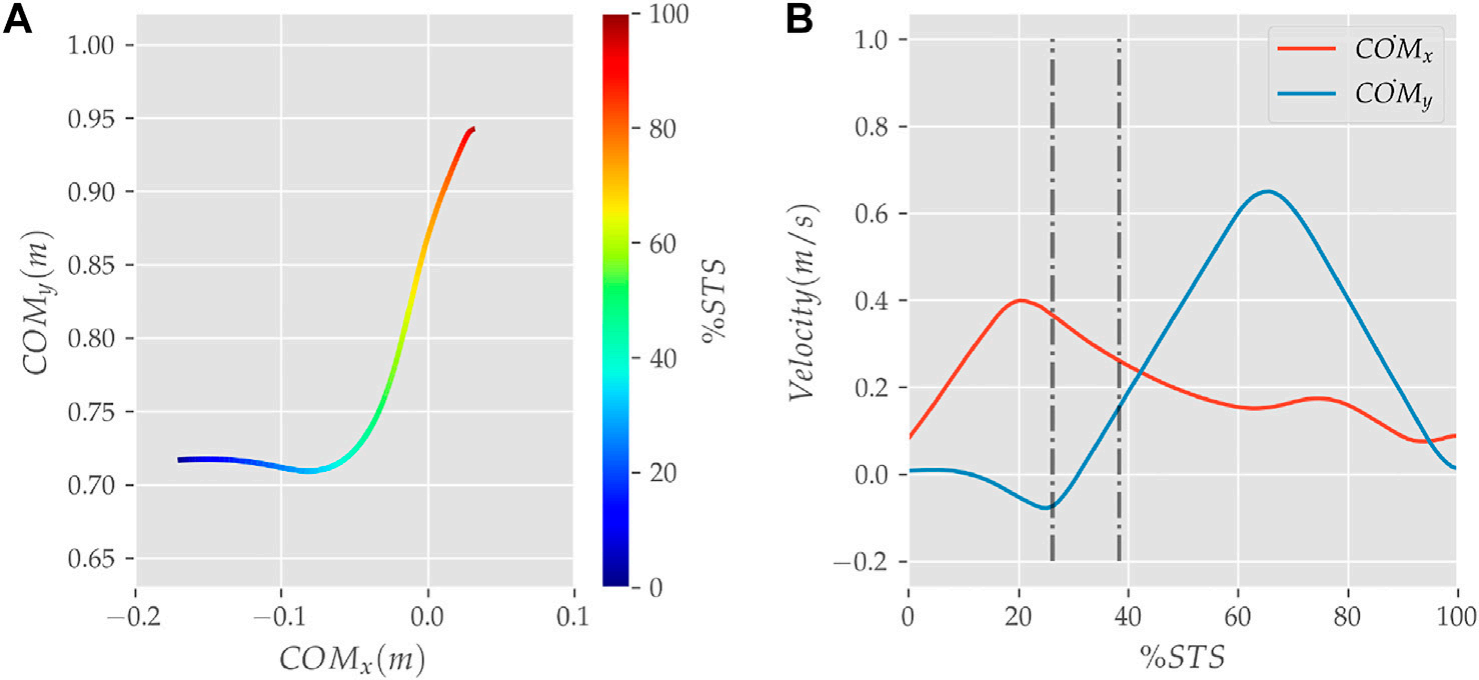
**(A)** Evolution of COM position and **(B)** velocity for the 0% strength deficit model’s STS transition.

**FIGURE 9:**
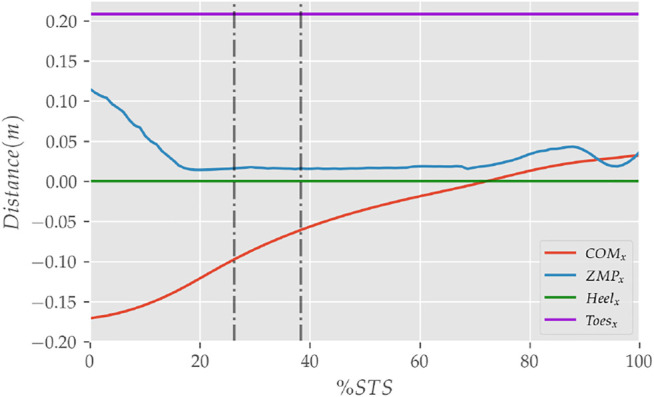
The zero moment point (feet forces) and the body’s COM trajectories from the 0% strength deficit model’s STS transition.

**FIGURE 10:**
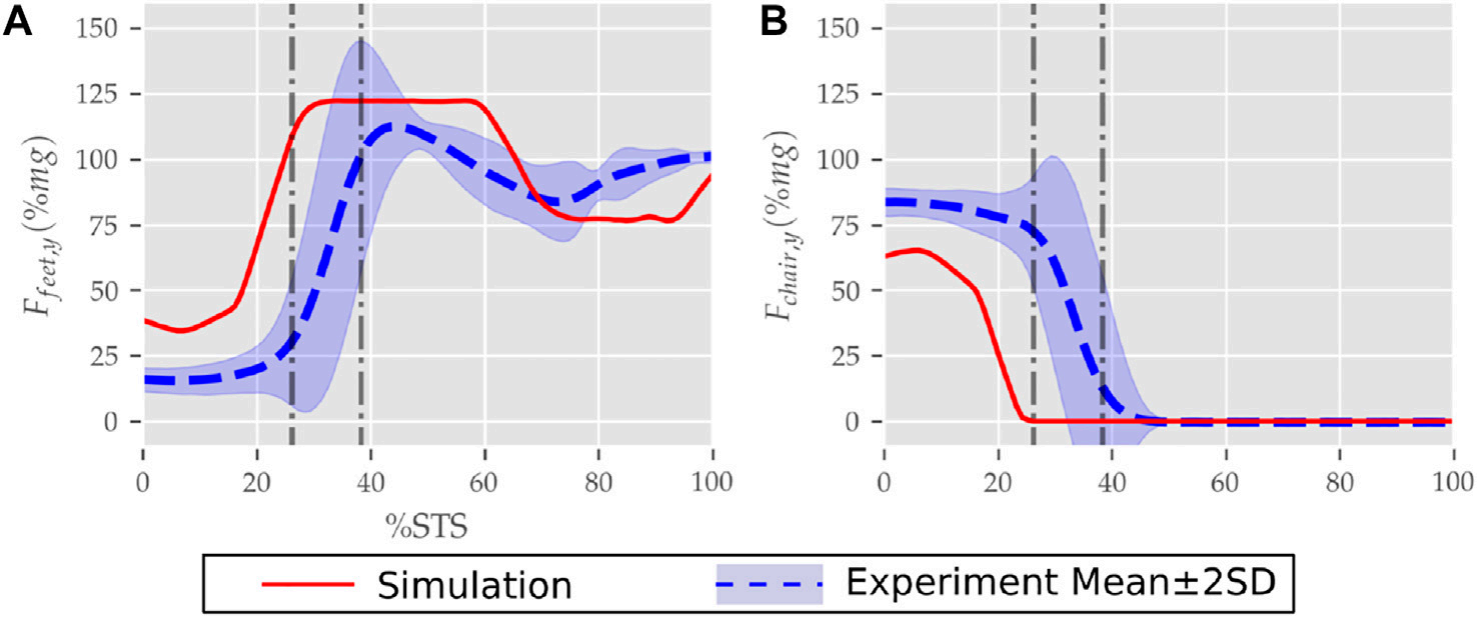
**(A)** Feet and **(B)** seat contact forces observed during the STS trajectory of the 0% strength deficit model and the experiments.

**FIGURE 11:**
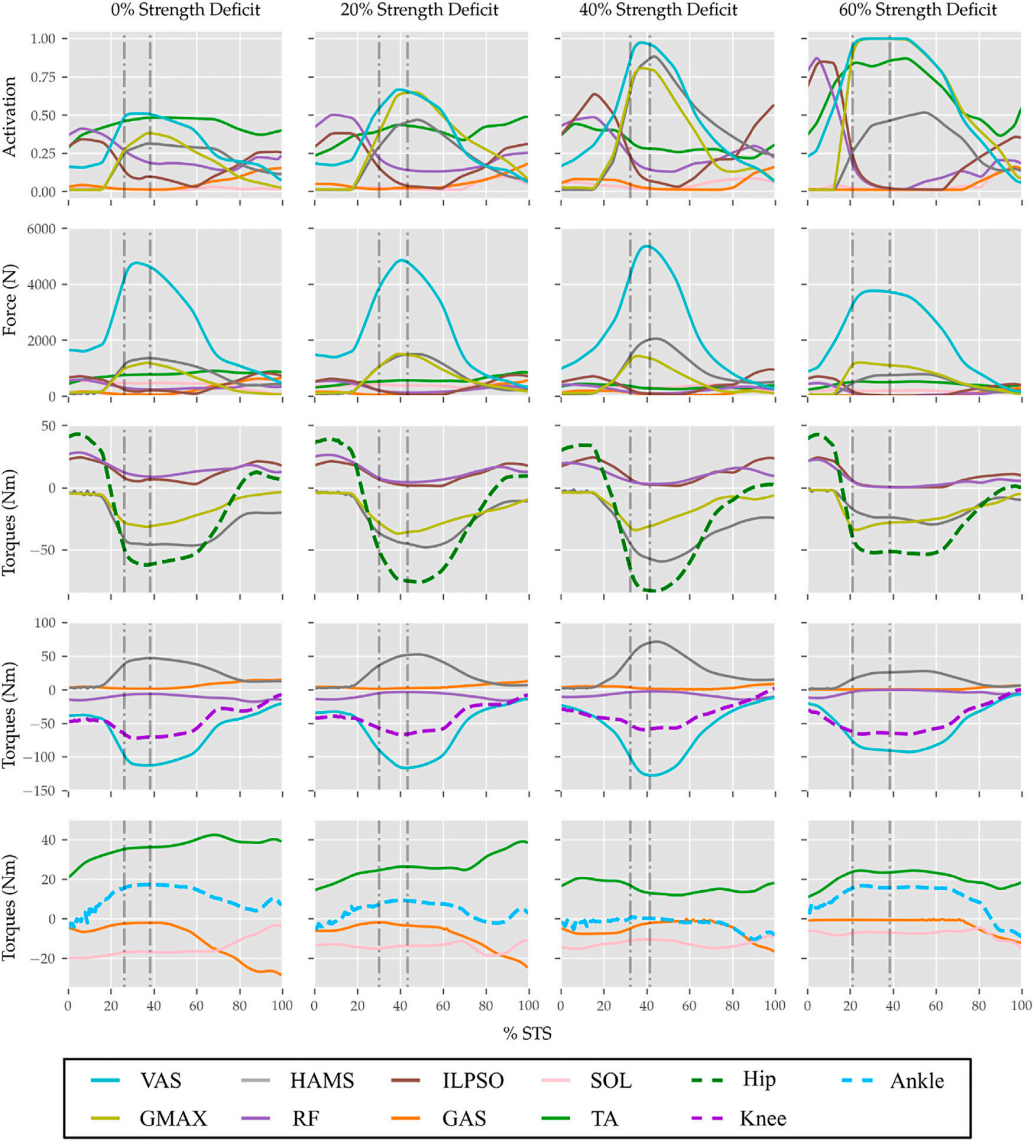
Muscle activations, muscle forces, and their respective contributions to the resultant joint torques from the STS trajectories of 0, 20, 40 and 60% strength deficit models.

**FIGURE 12:**
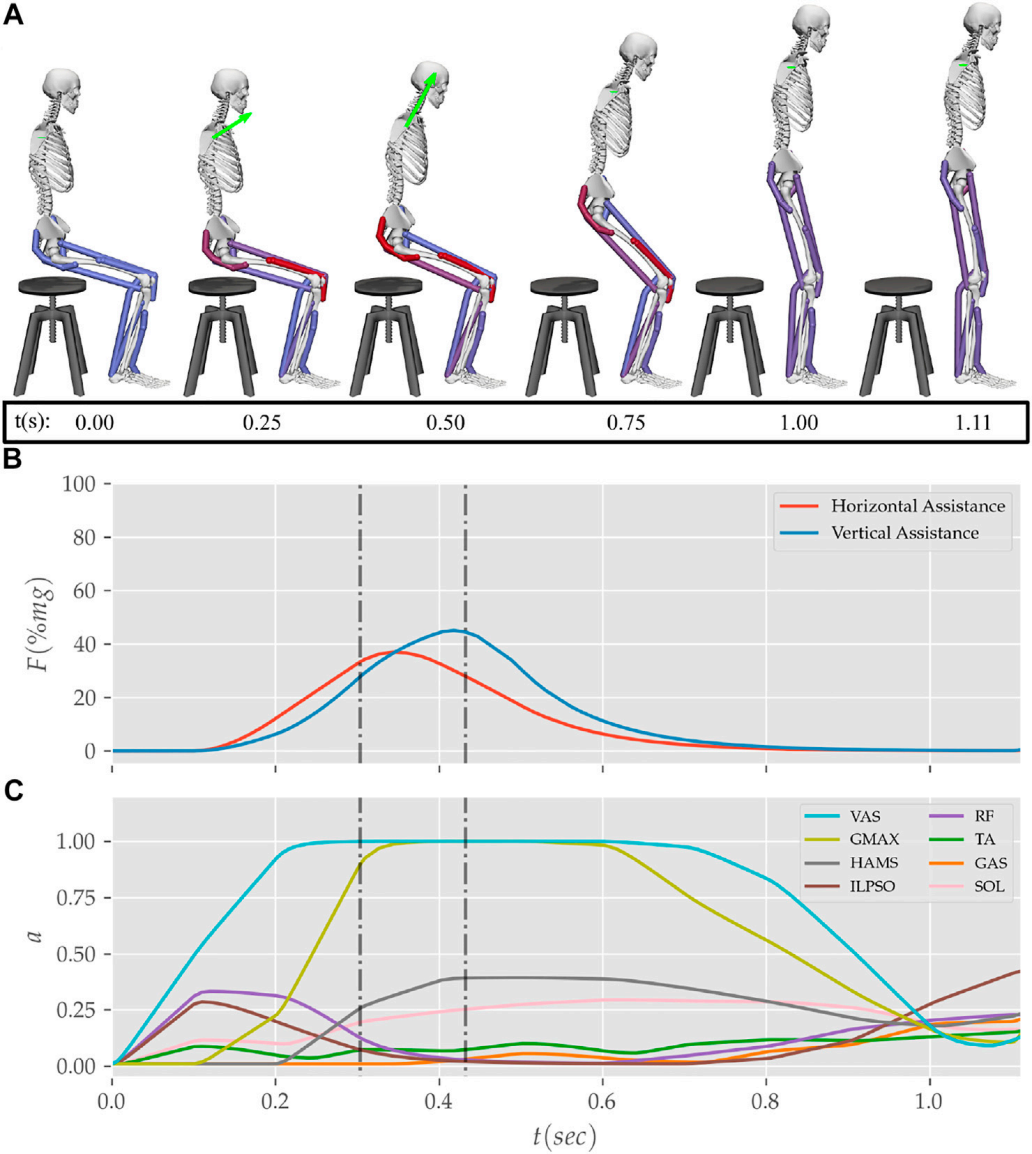
**(A)** Postures, **(B)** external assistance and **(C)** muscle activation trajectories from the STS transition of the externally assisted 80% strength deficit model. The green arrow in **(A)** represents the resultant external assistance force.

The optimization could generate successful STS trajectories for the 0, 20, 40 and 60% strength deficit models. However, for the 80% strength deficit model, the optimization could generate successful STS trajectories only when the model was assisted externally. The STS trajectories are divided into the three phases suggested in Millington et al. (1992) to facilitate discussions. Phase 1 starts with the trunk flexion and ends when the model loses contact with the chair. Phase 2 starts with the knee extension and ends when the hip joint is maximally flexed. Phase 3 begins with the reversal of trunk flexion to extension and ends with the model standing upright. The vertical black dotted lines in Figures 6–12 marks the transition between the three phases.

The results are organized into three subsections. In subsection 3.1 the kinematics and dynamics of the 0% strength deficit model’s STS trajectory are discussed and contrasted against the experimental observations. Subsection 3.2 details the adaptations and STS failure caused by muscle strength deficits. Subsection 3.3 discusses the features of the externally assisted 80% strength deficit model’s STS trajectory. Please refer to Figures 6–11; Table 4 during the following subsections for details. The resultant joint torques, in Figure 11; Table 4, were obtained using inverse dynamical analysis of the STS trajectories. During inverse dynamical analysis, the muscles forces were excluded, while the seat constraint and assistance forces were supplied as external forces. The resultant joint torques and the contributions of different muscles to them were computed using OpenSim (Delp et al., 2007).

**TABLE 4:** Properties of the 0, 20, 40, 60% and externally assisted 80% strength deficit model’s STS trajectories. Rows five, six, eight and nine show contributions of muscles to peak resultant joint torques.

# Row	Property	0*%* strength deficit	20*%* strength deficit	40*%* strength deficit	60*%* strength deficit	80% strength deficit assisted
1	STS duration (*s*)	1.14	1.23	1.33	1.47	1.11
2	Peak COM Horizontal Velocity (*m*/*s*)	0.40	0.42	0.42	0.39	0.43
3	Peak COM Vertical Velocity (*m*/*s*)	0.65	0.71	0.65	0.55	0.42
4	Peak Hip Torque (*Nm*)	−62.17	−76.10	−83.28	−53.58	−35.59
5	GMAX Peak Hip Torque (*Nm*)	−31.60	−35.12	−30.03	−26.17	−20.96
6	HAMS Peak Hip Torque (*Nm*)	−45.91	−47.15	−58.51	−29.31	−15.44
7	Peak Knee Torque (*Nm*)	−72.02	−66.73	−59.22	−65.86	−42.19
8	VAS Peak Knee Torque (*Nm*)	−111.26	−115.92	−125.02	−85.51	−42.36
9	HAMS Peak Knee Torque (*Nm*)	44.40	50.34	66.43	20.41	2.69
10	Peak VAS Force (*N*)	4754.10	4857.40	5355.19	3765.91	1907.14
11	Peak GMAX Force (*N*)	1194.27	1513.42	1437.11	1206.33	615.33
12	Peak HAMS Force (*N*)	1366.03	1505.99	2058.45	782.31	340.31

### 3.1 Unassisted STS Trajectory of 0% Strength Deficit Model

The joint angle, the muscle excitation, the COM position and velocity, the feet force zero moment point (*ZMP*), and the contact force trajectories associated with the 0% strength deficit model’s STS trajectory are respectively illustrated in Figures 6–10. The STS motion is initiated by activating the ILPSO and RF muscles (Figure 7). Their activation generates torque around the hip joint and flexes the torso forward (Figure 11). It is followed by the deactivation of ILPSO and RF muscles and gradually increasing activations of the GMAX and HAMS muscles. Due to the trunk’s forward flexion, the COM’s horizontal velocity increases and peaks (Figure 8) before the activations of the GMAX and HAMS muscles increase to control the torso’s forward flexion. Also, the activation of VAS muscle increases to prepare for seat-off. Phase 1 ends when the VAS muscle has generated sufficient torques around the knee joint to lift the musculoskeletal model off the chair. The seat off takes place with the body’s COM lying behind feet force ZMP (Figure 9). During phase 2, the GMAX and HAMS muscle activations increase until the hip flexion velocity reduces to zero. At this point, the trunk is maximally flexed, and phase 2 comes to an end. The knee joint extends only slightly during phase 2. The peak VAS, GMAX and HAMS muscle activations occur during phase 2. During phase 3, the activation of GMAX, HAMS, and VAS muscles slowly taper off because smaller forces are required to continue standing up due to an increasing fraction of body weight being borne by bone alignment. These patterns lead to the extension of both the hip and knee joints until the standing posture is achieved. At the end of phase 3, increased activation is observed in ILPSO, RF, and TA muscles to stop the hip, knee and ankle joints from extending past the upright posture. Also, during the latter half of phase 3, the body’s COM reaches the feet support polygon. The SOL muscles see almost negligible activation; however, it produces significant passive fiber forces during the first two phases and a significant part of the third phase. Significant TA muscle activations are present during all three phases. These activations produce the force needed to balance the counteracting SOL and GAS muscle forces.

The joint angle trajectories of 0% strength deficits are contrasted against those observed experimentally for a healthy adult in Figure 6B. The general shape of the hip and knee joint angle trajectories matches those of experiments. The discrepancies in the joint angle trajectories primarily result from the different initial postures (Figure 5). The mean initial posture from experiments requires the lumbar joint extension from −30° to nearly 0°. Our model did not include lumbar joint actuation for the reasons of modelling simplification. The initial posture was modified to compensate for the non-actuated lumbar joint by moving the model slightly forward and locking the lumbar joint with 10° of flexion.

The muscle activation patterns of the 0% strength deficit model’s STS trajectory are compared to those of experiments in Figure 7. The general shape of activation patterns for the STS critical muscles, i.e., GMAX, HAMS and VAS, matches the experiments. The higher activation of VAS muscle than experiments during the first half of phase 1 is potentially due to cost term *ϕ*_2_. Muscle RF features higher activation during STS initiation as the model did not feature trunk muscles. The higher activation of TA muscle than experiments is potentially due to the passive fiber forces induced in the SOL muscle by the initial posture. Experimental data features a small peak in the TA, GAS and SOL muscle activations during phase 2. This peak is absent in the generated STS. The experimental data did not include EMG signal for ILPSO muscle. The peak activations of all the muscles except RF and TA are within the two standard deviations of the peaks observed experimentally.

The seat and feet contact force trajectories of the 0% strength deficit model’s STS transition are compared to the experimental observations in Figure 10. The lower seat-pan forces than experiments are most potentially because of the point on point constraint-based formulation. The flattening in the peak feet forces for simulation is because of the cost term *ϕ*_10_ and the absence of control noise. Also, the seat-off in simulation occurs earlier than in the experiments because the simulation’s initial posture requires less horizontal momentum to stand up, and the kinematic constraint-based seat force formulation makes its development easier.

### 3.2 STS Adaptations and Failure

With strength deficits, the STS duration and the peak VAS, GMAX, RF, ILPSO and TA muscle activations increase (Table 4; Figure 11). The peak HAMS muscle activation increases with muscle weakness up to 40% and then decreases for the 60%. The peak VAS muscle activation is higher than that of GMAX muscle up to 40% strength deficits and is equal for the 60% strength deficit. The decrease in the peak HAMS muscle activation from 40 to 60% strength deficit is to alleviate the saturated VAS muscle antagonistic at the knee joint. It is evident from the contribution of HAMS muscle to peak resultant knee torques dropping from −112.17*%* for the 40% strength deficit to −30.99*%* for the 60% strength deficit. The reduced HAMS muscle activation saturates the GMAX muscle as they work together to control the hip flexion. It is demonstrated by the contributions of HAMS muscle to the peak resultant hip torques dropping from 70.26*%* for the 40% strength deficit to 54.7*%* for the 60% strength deficit. Also, a reduction in the peaks of COM velocity, ground reaction forces, and GMAX, HAMS and VAS muscle forces is observed from the 40–60% strength deficits. Bobbert et al. (2016) also observes that with strength deficits, the STS duration increases, while the peak COM vertical velocity, peak GMAX, and VAS muscle forces decrease. However, Bobbert et al. (2016) does not observe any significant reduction in HAMS muscle activation. It is potentially because Bobbert et al. (2016) used the immediately prior solutions as the initial guess for the subsequent optimization. Besides STS duration and peak muscle activation, we do not observe consistent trends from the 0–40% strength deficits. It is most potentially because the optimizations converged to different locally optimal solutions for each model.

The optimization framework failed to generate STS transitions using the 80% strength deficit model. We suspected the GMAX or the VAS muscle to be responsible for this failure as they were getting saturated for the 60% strength deficit model’s STS trajectory (Figure 11). We tracked the 60% strength deficit model’s successful STS trajectory using the 80% strength deficit and two different reserve actuator setups. In the first setup, the optimal torque, i.e., torques generated per unit control effort, for the hip and knee torque actuators were 100*Nm* and 1*Nm* respectively, while for the second setup, they were 1*Nm* and 100*Nm*. The first setup favored the utilization of the hip reserve actuator, while the second setup favored the utilization of the knee reserve actuator. The first setup’s motion-tracking features a peak torque of −19.81*Nm* by the knee reserve actuator and increased activation of both VAS and RF muscles. The second setup-based motion-tracking features a peak torque of −12.05*Nm* by the hip reserve actuator and increased HAMS and GMAX activations. The lower magnitude of reserve actuator in the second setup suggests that the STS failure occurred because of VAS muscle weakness. Also, the observation that peak VAS muscle activation is greater than or equal to that of GMAX muscle supports this hypothesis.

### 3.3 Externally Assisted STS Transition

During the motion tracking of the previous subsection, it was observed that assisting the musculoskeletal model primarily at the hip joint lead to increased RF muscle activation, while assisting it primarily at the knee joint lead to increased HAMS muscle activation. As STS transition is performed several times a day, assisting only at the hip or the knee joint has a high potential to cause the RF or the HAMS muscle contracture. Both the muscles cross the hip joint, and their contracture can cause back pain issues if not diagnosed. Thus the external assistance was introduced at the torso COM in the 80% strength deficit model. Also, assisting the model at the torso center of mass is a good approximation for assisting a human at the underarms area. The underarms area is easily graspable, and assistance using it helps simplify the design of probable STS assistance devices.

Physical assistance can help maintain or recover lower extremity strength when provided in an assist-as-needed manner. Thus while generating the assisted STS trajectories, the over-utilization of external assistance was penalized (Eq. 6). Figure 12 shows the body postures, the assistance forces, and muscle activation for the externally assisted 80% strength deficit model’s STS trajectory. The trajectory features utilization of external assistance when the VAS and GMAX muscle starts getting saturated, i.e., the model uses external assistance only when needed. The peak magnitudes of external assistance’s vertical and horizontal components are 36.50 and 44.51*%* of the body’s weight. The STS trajectory features reduced peaks of COM velocities, resultant hip and knee joint torques and the VAS, GMAX, and HAMS muscle forces. The seat-off takes place with the torso more upright than unassisted models.

## 4 Discussion

This paper presented and analyzed the sit-to-stand (STS) trajectories generated using an open-loop single shooting optimization and musculoskeletal models with different strength deficits. The strength deficits were introduced by simultaneously scaling the maximum isometric strength of all the muscles in steps of 20%. The optimization could successfully generate STS trajectories for models with up to 60%strength deficits. The muscle activation patterns for the 0% strength deficit model agree reasonably with the experimental observations for a healthy adult. A reduction in the peak HAMS muscle activation is observed when the VAS muscle, antagonistic across the knee joint, gets saturated due to the strength deficits. The reduced HAMS muscle activation saturated the GMAX muscle. After clinical validation, the reduced ratio of peak HAMS to GMAX muscle activation can be used to plan intervention. Then, the motion-tracking results were used to suggest the VAS muscle weakness to be responsible for optimization’s failure to generate STS trajectories using the 80% strength deficit model. The motion tracking results were also used to motivate the introduction of external assistance at the torso’s centre of mass (COM). The optimization could generate successful STS trajectories for the externally assisted 80% strength deficit model. The optimal trajectory featured the utilization of external assistance in an assist-as-needed manner. We have made the source code for optimization public to speed up the design of future assist-as-needed STS care devices. Finally, the findings of this study should be observed with caution as they have many inherent assumptions. The most significant among them are discussed in the following next paragraphs, followed by our probable future research directions.

Many experimental studies report that the elderly follow a stabilization strategy in which they move the body’s COM over the feet support polygon before getting off the chair. Like the mean initial posture of our experiments, the stabilisation strategy requires significant lumbar motion. For our musculoskeletal model, the body’s COM lies just 1.15 *cm* inside the feet support polygon when the trunk is maximally flexed while maintaining chair contact. Thus the elimination of the lumbar joint and the feet-ground relative degree of freedom, even though also made by Pandy et al. (1995), Bobbert et al. (2016), and Yokota et al. (2016), might have been oversimplifications for predicting STS trajectories of the elderly adults.

The strength deficits were introduced by simultaneously scaling all the muscles’ maximum isometric strength. However, the strengths of all the muscles do not deteriorate by the same ratio. Also, scaling the maximum isometric forces is not the only way to introduce strength deficits. For example, the peak muscle activations could have been limited to the same effect. Thus the strength deficit modelling, even though made similarly by Bobbert et al. (2016) and Yokota et al. (2016), should be investigated for more accurate predictions.

We assumed a sagittal plane of symmetry. However, it has been shown that even for healthy adults, one leg is usually more dominant than the other. Also, significant asymmetries may arise when one of the upper extremities grabs surfaces for assistance. Thus, the optimization framework needs to be extended to use the 3D musculoskeletal model to generate more realistic assisted and unassisted STS trajectories. Other musculoskeletal model-related critical assumptions that must be validated are simplifying the muscle groups to single musculotendon units and the control level decoupling of muscles.

Perfect coordination between the musculoskeletal model and the external assistance was assumed. It led to an optimal assisted STS transition with 1.11*sec* STS duration and is unrealistic to replicate. The optimization framework should be extended to include sensory noise and delay in external assistance formulation to synthesize realistically replicable STS trajectories. The maximum simulation duration needs to be extended beyond 1.6*sec*. The chair height and the initial posture heavily influence the STS transitions, and the results of this study are a function of them.

The cost function used in this study is not unique in its capability to engender STS. Further, even for the selected cost function, the relative weights of the different cost terms should have been chosen using inverse optimal control. The relative weights were selected using trial and error because of the computationally demanding nature of the optimization. The generated STS trajectories are local optimal solutions of nonlinear non-convex optimizations. The optimization’s failure to generate STS using the 80% strength deficit model might have been due to the unsuccessful search rather than muscle saturation.

We plan to design a kinematic events-based closed-loop STS controller in the future. We also plan to investigate the torque and muscle actuated lumbar joint models for STS trajectories with more accurate joint kinematics and dynamics. Finally, we intend to extend the optimization framework to include sensory noise and delay for the more realistic models of assist-as-needed STS care devices.

## Data Availability

The source code and the data used in this study are available at https://github.com/ShibataLab/PredictiveSTS.
