# Supplementary material for: Predicting Sit-to-Stand Adaptations due to Muscle Strength Deficits and Assistance Trajectories to Complement Them
Source: Front Bioeng Biotechnol. 2022 Mar 18;10:799836. doi: 10.3389/fbioe.2022.799836 (PMC8971612; doi:10.3389/fbioe.2022.799836)
Supplement: Supplementary file 1 [file DataSheet1.pdf]

frontiers

Supplementary Material

1 SUPPLEMENTARY FIGURES

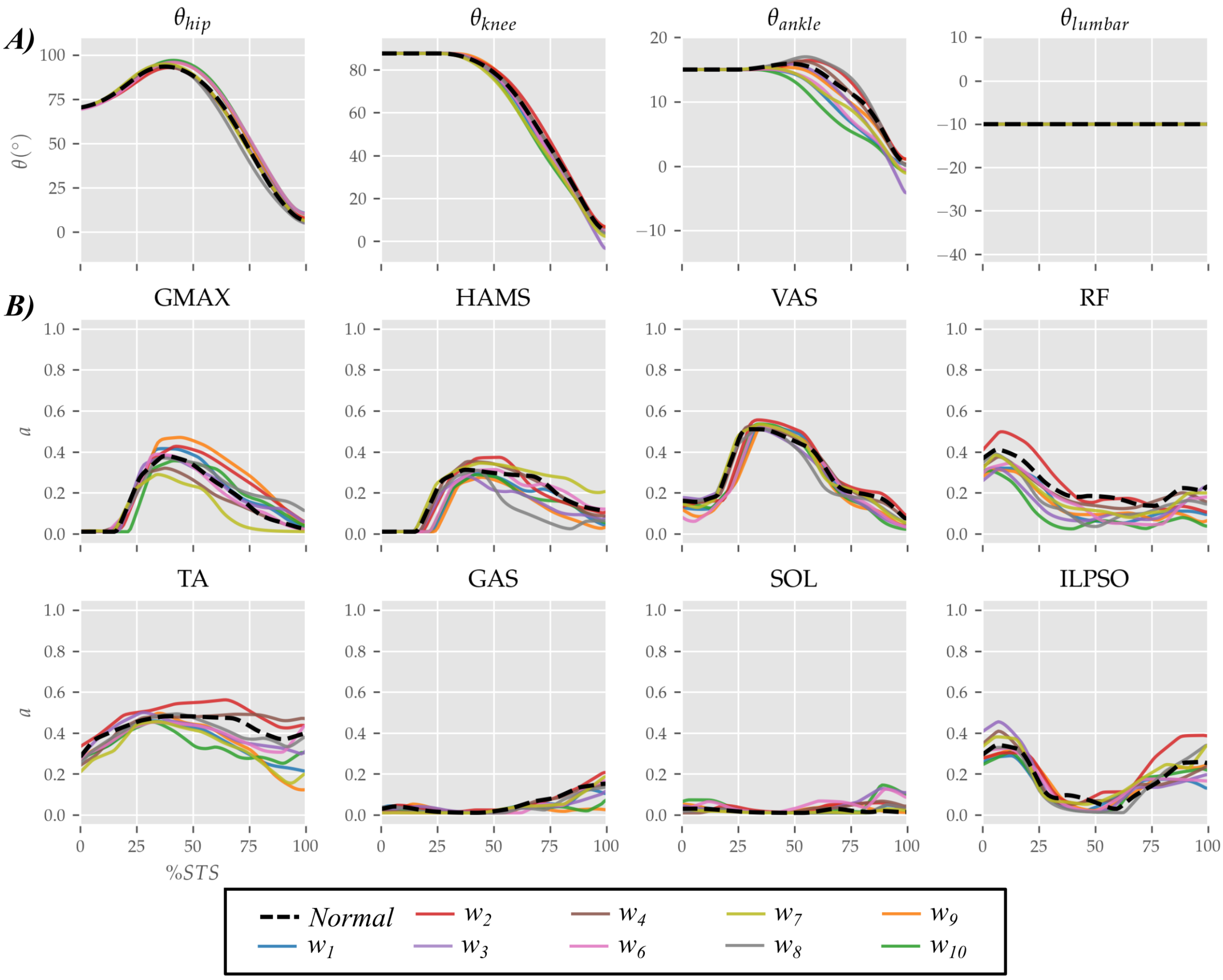

Figure S1. Joint angle **(A)** and muscle activation **(B)** trajectories obtained using the 0% strength deficit model with normal relative weights as listed in Table 3 and the relative weights increased individually by 10%. The resulting STS trajectories appear reasonably robust to relative weights.

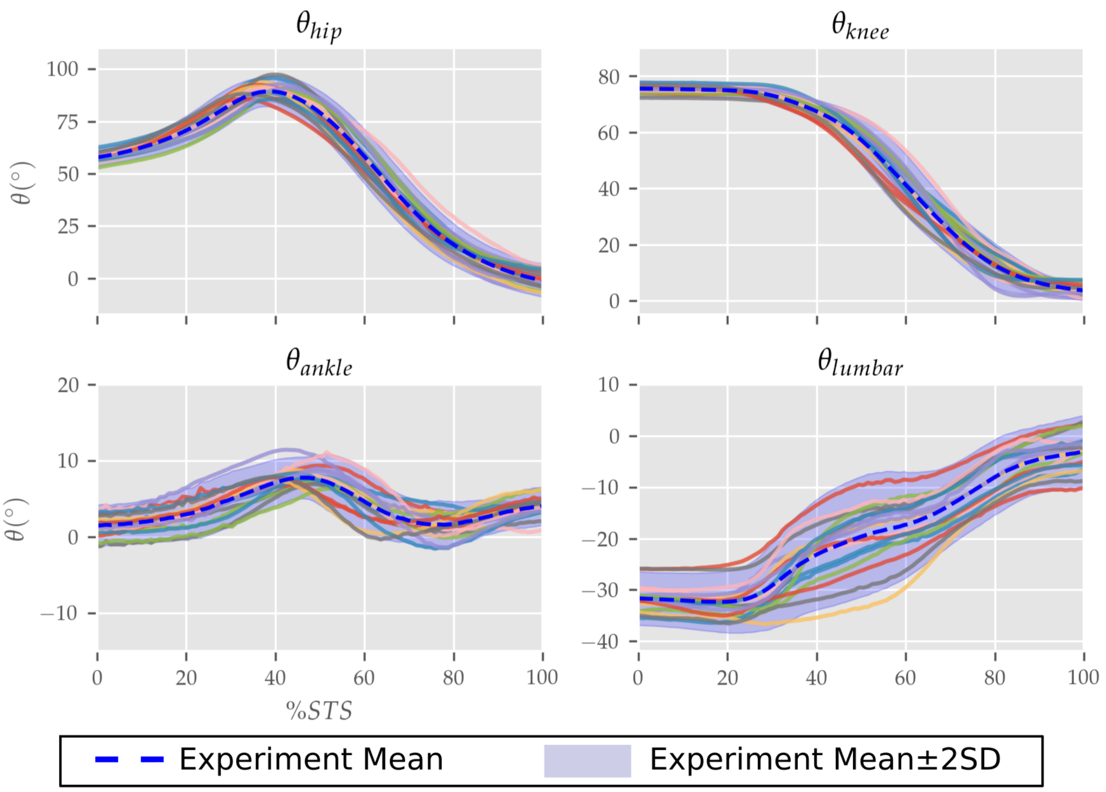

Figure S2. Joint angle trajectories from the experimental trials of the healthy adult. The beginning and the end of STS were defined as the points when hip flexion and hip extensions velocities smoothed with a rolling window of $0.1s$ grew respectively higher or lower than $20°/s$.

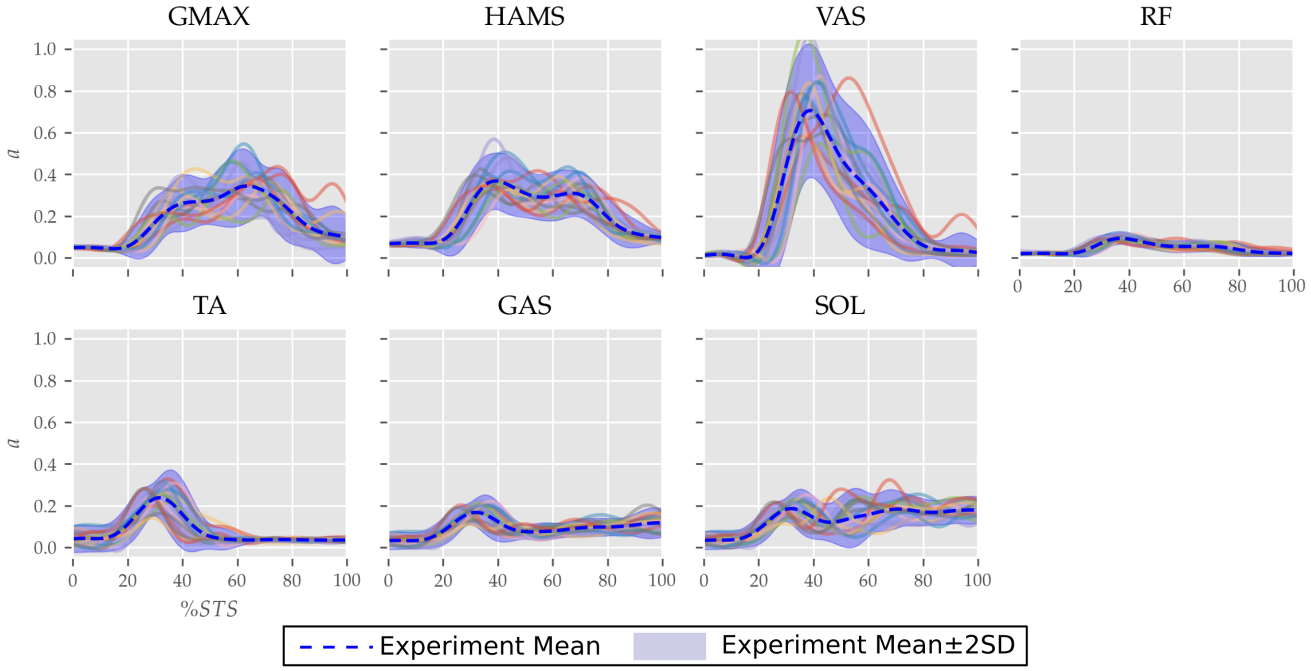

Figure S3. Muscle activation trajectories from the experimental trials of the healthy adult.

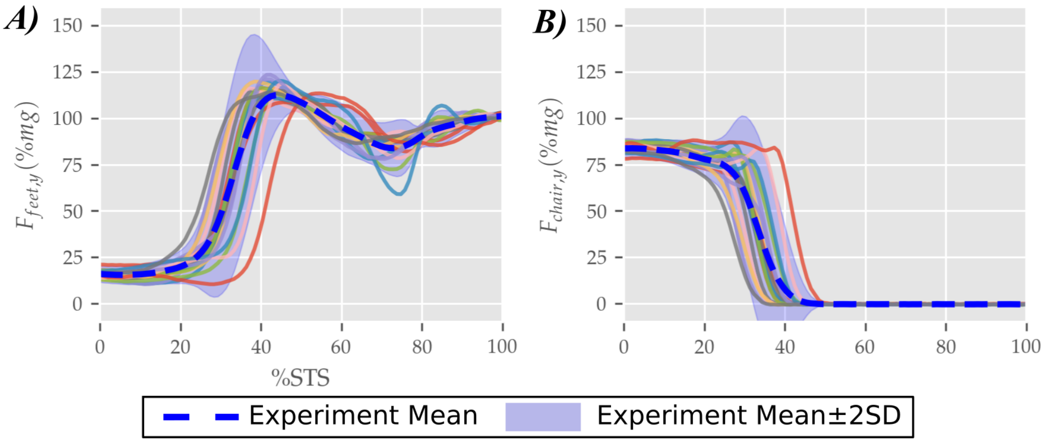

Figure S4. Seat and ground reaction force trajectories from the experimental trials of the healthy adult.
